# The ROP2 GTPase Participates in Nitric Oxide (NO)-Induced Root Shortening in Arabidopsis

**DOI:** 10.3390/plants12040750

**Published:** 2023-02-08

**Authors:** Erzsébet Kenesi, Zsuzsanna Kolbert, Nikolett Kaszler, Éva Klement, Dalma Ménesi, Árpád Molnár, Ildikó Valkai, Gábor Feigl, Gábor Rigó, Ágnes Cséplő, Christian Lindermayr, Attila Fehér

**Affiliations:** 1 Institute of Plant Biology, Biological Research Centre, Eötvös Lóránd Research Network, Temesvári Krt. 62, H-6726 Szeged, Hungary; 2 Department of Plant Biology, University of Szeged, Közép Fasor 52, H-6726 Szeged, Hungary; 3 Laboratory of Proteomics Research, Biological Research Centre, Eötvös Lóránd Research Network, Temesvári Krt. 62, H-6726 Szeged, Hungary; 4 Hungarian Centre of Excellence for Molecular Medicine, Single Cell Omics ACF, H-6728 Szeged, Hungary; 5 Institute of Biochemical Plant Pathology, Helmholtz Zentrum München—German Research Center for Environmental Health, Ingolstädter Landstraße 1, D-85764 Neuherberg, Germany

**Keywords:** *Arabidopsis thaliana*, auxin transport, post-translational modification, PIN1, Rho-type small GTPase, root meristem, S-nitrosation

## Abstract

Nitric oxide (NO) is a versatile signal molecule that mediates environmental and hormonal signals orchestrating plant development. NO may act via reversible S-nitrosation of proteins during which an NO moiety is added to a cysteine thiol to form an S-nitrosothiol. In plants, several proteins implicated in hormonal signaling have been reported to undergo S-nitrosation. Here, we report that the Arabidopsis ROP2 GTPase is a further potential target of NO-mediated regulation. The ROP2 GTPase was found to be required for the root shortening effect of NO. NO inhibits primary root growth by altering the abundance and distribution of the PIN1 auxin efflux carrier protein and lowering the accumulation of auxin in the root meristem. In *rop2-1* insertion mutants, however, wild-type-like root size of the NO-treated roots were maintained in agreement with wild-type-like PIN1 abundance in the meristem. The ROP2 GTPase was shown to be S-nitrosated in vitro, suggesting that NO might directly regulate the GTPase. The potential mechanisms of NO-mediated ROP2 GTPase regulation and ROP2-mediated NO signaling in the primary root meristem are discussed.

## 1. Introduction

The development of the plant roots is plastic to allow changes in root system architecture (RSA), ensuring adaptation to nutrient and water availability, as well as to unfavorable soil conditions [1,2,3,4,5]. RSA, the spatial configuration of the entire root system in the soil, depends on the positioning, orientation, growth, and branching of various root types [6]. The elongation of the primary root (PR), similarly to many other plant developmental processes, is regulated mainly by auxin in cooperation with other signaling molecules [7,8,9]. Along the Arabidopsis main root, there is an auxin gradient due to directional shoot-to-root auxin transport in the vascular parenchyma [10,11], as well as to local synthesis in the meristem [12]. Auxin accumulates in the highest quantity in the root meristem, where it maintains the cell division activity of stem cells [7,10,11].

The key role of NO as a gaseous signal molecule was demonstrated to modulate hormonal crosstalk during various developmental or stress-induced alterations of RSA (for reviews [13,14,15]). NO treatment can evoke morphogenic responses in roots reducing the length of the PR [16], but enhancing lateral [17] or adventitious [18] root formation and root hair growth [19]. The effect of NO on the RSA reflects common morphogenic changes evoked by various stress treatments, the so-called “stress-induced morphogenic response” (SIMR) [20]. Auxin has a central role in SIMR [21], and in addition to ethylene and reactive oxygen species [22], NO was also implicated in the process [23]. NO and auxin have an intricate relationship during SIMR; NO has been proven to have a downstream effector as well as an upstream regulator role in auxin responses [24,25,26]. In one hand, exogenous NO causes root meristem defects due to reduced PIN1-mediated acropetal auxin transport [16]. On the other hand, depletion of NO perturbs auxin biosynthesis, transport, and signaling, resulting in small root meristems with abnormal cell divisions and stem cell niche organization [26].

The direction of auxin flow is primarily determined by the polar localization of PIN efflux carriers in the membrane of competent auxin transport cells [27]. PIN1 mainly resides in the basal membrane of vascular parenchyma cells and contributes to the accumulation of shoot-derived auxin in the root meristem [10]. Polar accumulation of PIN proteins at specific membrane domains is a dynamic process [28,29]. Endocytic recycling of PIN proteins to prevent lateral diffusion from the site of polarity is controlled by Rho-of-plant (ROP) GTP-binding proteins [30]. ROP G-proteins are central regulators of cell polarity in plants [31,32]. They cycle between a GTP-bound active and a GDP-bound inactive conformation is fine-tuned by regulatory proteins: the guanine nucleotide exchange factors (GEFs), the GTPase-accelerator proteins (GAPs), and the guanine nucleotide dissociation inhibitors (GDIs) [33]. In the GTP-bound state, they bind various downstream effectors mediating cell-polarity establishment [34,35]. These effectors, among others, have a role in modifying auxin transport. The jigsaw puzzle-like leaf epidermal cell shape is regulated by ROP2 signaling that inhibits PIN1 endocytosis stabilizing PIN1 polarization at the lobe regions of pavement cells [36]. In the root meristem, the auxin-activated ROP6 pathway was found to regulate PIN1 and PIN2 endocytosis in the stele and cortex/epidermal cells, respectively [30,37,38].

NO affects cellular processes primarily via the direct posttranslational modification of proteins. The bioactivity of NO can be exerted through protein S-nitrosation, a process during which an NO moiety is added to a cysteine thiol (–SH) to form an S-nitrosothiol (SNO) [39]. S-nitrosation might control protein function and regulation, interfering with other posttranslational modifications, such as phosphorylation, SUMOylation, acetylation, etc. [40]. Protein S-nitrosation has already been shown to play a role in NO-auxin interaction [41,42]. Among others, it was shown that NO causes S-nitrosation of the auxin receptor TIR1/AFB proteins increasing auxin sensitivity [42]. In animal cells, several small GTP-binding proteins of the Ras superfamily, including the Rho-type GTPases, are also S-nitrosation targets [43]. S-nitrosation of specific cysteine residues might alter the biochemical properties of Rho-type GTPases. Reactive nitrogen species (RNS) were found to affect the nucleotide binding of human Rac1, RhoA, and Cdc42 GTPases in vitro [44] or in vivo [43,45]. Despite the fact that the S-nitrosation-sensitive GXXXXGK(S/T)C P-loop motif is highly conserved in all the Rho-type GTPases, including the ones in plants, there is no report investigating this type of potential posttranslational regulation of ROPs.

Here, we report that the Arabidopsis ROP2 GTPase is required for exogenous NO-dependent root growth inhibition. The Arabidopsis *rop2-1* mutant proved to be insensitive to exogenous NO considering PR elongation. This insensitivity correlated with PIN1 abundance in the mutant and the wild-type-root tip. The potential mechanisms of ROP2 GTPase mediated NO action on PIN1 abundance are discussed with a focus on potential in planta ROP2 S-nitrosation affecting its cellular distribution.

## 2. Results

### 2.1. The Roots of Arabidopsis rop2-1 Mutant Seedlings Are Less Sensitive to Exogenous NO Than That of the Wild Type

The ROP2 Arabidopsis GTPase has been implicated in the control of PIN1 polarity [30]. To test its potential role in the root-shortening effect of exogenous NO, 7-day-old Arabidopsis wild type (Col-0), ROP GTPase mutant (*rop2-1*; [46]), and pROP2:GFP:ROP2 complemented *rop2-1* mutant [47] seedlings were exposed to increasing concentrations of the NO donors GSNO or SNAP.

**Figure 1:**
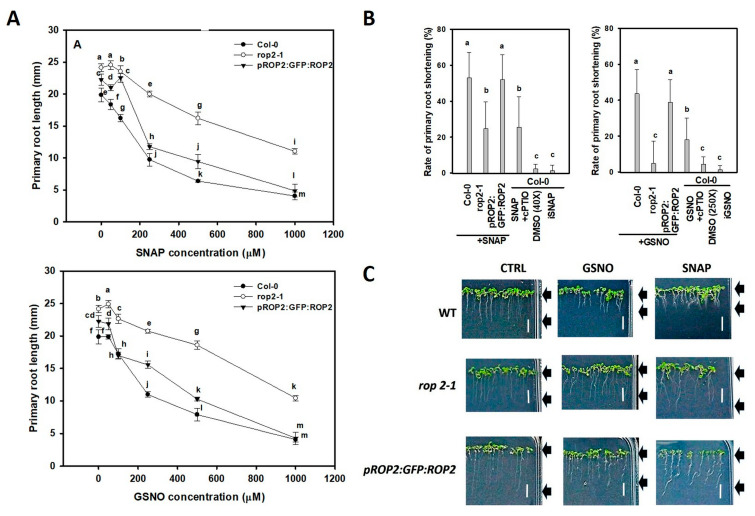
(**A**) Primary root length (mm) of Col-0, *rop2-1*; and pROP2:GFP:ROP2 complemented *rop2-1* (labelled as pROP2:GFP:ROP2) Arabidopsis lines treated with 0, 50, 100, 250, 500, or 1000 µM SNAP or GSNO for 72 h. (**B**) Percentage values of primary-root shortening induced by 250 µM SNAP or GSNO in the investigated lines of Arabidopsis. Appropriately diluted DMSO, light-inactivated NO donors (iSNAP and iGSNO), and 250 µM SNAP/GSNO plus 800 µM cPTIO served as control treatments. (**C**) Images of seedlings of the same Arabidopsis lines treated with 250 µM SNAP or GSNO for 72 h. Arrows indicate the approximate length of the roots. CTRL is the control treated only with the solvent (DMSO in 250-fold and 40-fold dilutions for GSNO and SNAP, respectively). Bar = 10 mm. Data in (**A**,**B**) are averages and standard errors from three independent experiments with at least 10 roots each. Multiple comparison analyses were performed with SigmaStat 12 software using analysis of variance and Duncan’s test. Different letters sign significant difference at the *p* < 0.05 level.

It was observed that in the presence of NO donors, the PR length of all the investigated lines decreased in a dose-dependent manner (Figure 1A). At 250 µM GSNO or SNAP, there was an obvious difference in the NO sensitivity of the various genotypes. Although elevating the concentration of any of the two NO donors (500–1000 µM) further reduced the root length, it did not increase the difference among the lines, likely reflecting general toxic effects (Figure 1A). Therefore, in the further experiments, either 250 µM GSNO or SNAP was used. Endogenous NO levels (determined as the pixel intensity of DAF–FM-derived fluorescence) were found to be transiently increased in the root tips of wild-type Arabidopsis seedlings treated with 250 µM SNAP or GSNO, respectively. After 24 h of treatment, both NO donors caused a ca. three-fold rise in the root NO content as compared to untreated controls (Supplementary Figure S1). This was decreased near to the control level after 48 h (Supplementary Figure S1). NO liberation in GSNO and SNAP solutions was also quantified by an NO-specific electrode during similar circumstances to those of the plant treatments. In the 250 µM GSNO solution, the highest NO concentration (224.00 ± 5.29 nM) was observed after 15 min and remained higher than 100 nM until the 180 min of the measurement. From 250 µM SNAP, the concentration of liberated NO was the highest (626.65 ± 8.06 nM) after 30 min, and SNAP generated a constantly high amount of NO during the period of measurement (Supplementary Table S2).

At the 250 µM exogenous NO donor concentrations, the wild-type seedlings had a ca. 50% reduction in PR length, while the *rop2-1* mutant line exhibited only a ca. 20–25% (SNAP) or ca. 5–12% (GSNO) reduction (Figure 1B,C). In summary, the *rop2-1* mutant exhibited reduced sensitivity towards the PR-shortening effect of GSNO or SNAP in comparison to the wild type. The PR growth-reducing effect of both GSNO and SNAP was ameliorated by the addition of cPTIO and could not be observed applying light-inactivated NO donors (iSNAP, iGSNO) or the solvent (DMSO) alone (Figure 1B). These observations support the NO-specific effect of the applied GSNO and SNAP treatments. Complementation of the *rop2-1* mutation with a pROP2:GFP:ROP2 construct (designated as pROP2:GFP:ROP2) resulted in wild-type-like root shortening response to exogenous NO (Figure 1). This strengthened the specific role of the ROP2 GTPase mediating exogenous NO action on PR shortening. To further verify this specificity, seedlings of the *rop6-2* mutant of Arabidopsis were treated by 250 µM GSNO. The absence of ROP6, unlike that of ROP2, did not interfere with GSNO action (Supplementary Figure S2A). 

### 2.2. GSNO Promotes In Vitro S-nitrosation of the ROP2 Protein

**Figure 2:**
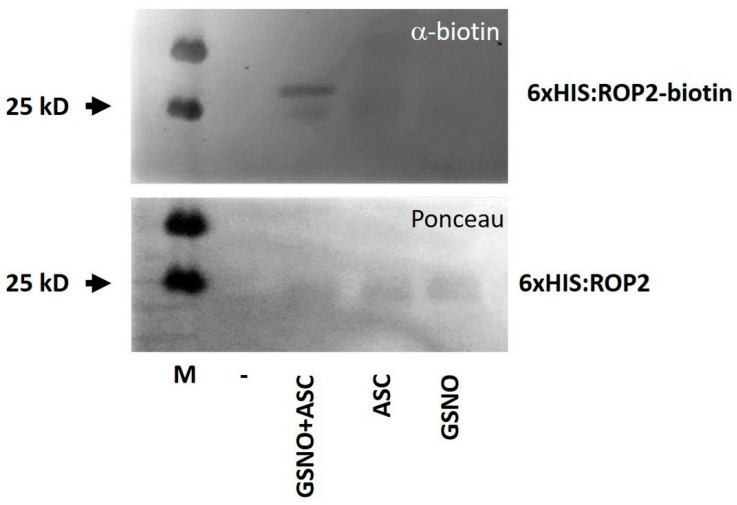
In vitro S-nitrosation of GSNO-treated 6xHIS:ROP2 protein as analyzed by Western blotting using an anti-biotin antibody (α-biotin) following the “biotin switch” method (upper picture). The absence of ascorbate (Asc) served as negative control. Ponceau S staining is shown as loading control (lower picture). M—molecular mass standard.

To examine the possibility that exogenous NO influences PR shortening via the post-translational modification ROP2, recombinant ROP2 protein was exposed to GSNO to investigate its potential S-nitrosation. 6xHIS-tagged ROP2 proteins were produced in and affinity purified from bacterial cells. The protein was subjected to 250 µM GSNO treatment, and S-nitrosated cysteines were converted to biotinylated cysteines followed by the detection of the biotinylated proteins by immunoblotting (“biotin switch”). The ROP2 protein produced a strong signal in the combined presence of GSNO and ascorbate (Asc), but only a low signal in response to GSNO or Asc alone, indicating S-nitrosation (Figure 2). Considering the conserved structure of ROP proteins, the in vitro nitrosation of ROP6 by GSNO was also tested and confirmed (Supplementary Figure S2A).

### 2.3. S-nitrosation Might Alter the Intracellular Localisation of ROP2

**Figure 3:**
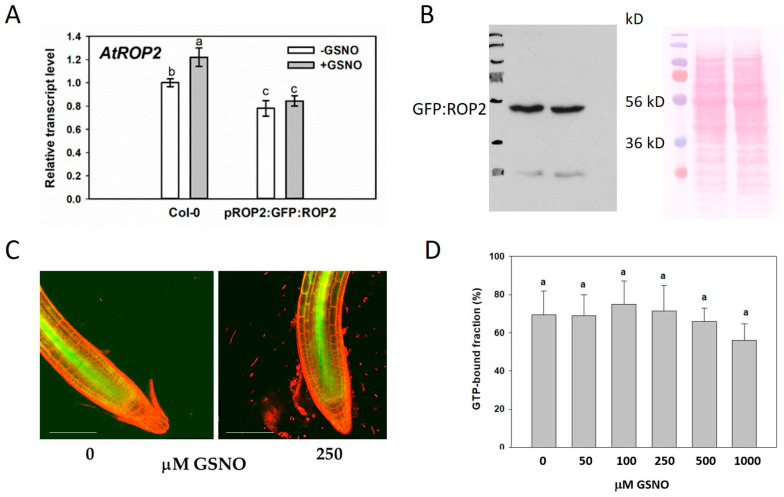
(**A**) Relative transcript level of ROP2 in control and 250 µM GSNO-treated Col-0 and pROP2:GFP:ROP2 Arabidopsis. Data were normalized using the A. thaliana *ACTIN2* and *GAPDH2* genes as internal controls. The relative transcript level in Col-0 control samples was arbitrarily considered to be 1. Data were averaged from three biological repetitions with three technical replicates each. (**B**) Western blot analysis of GFP:ROP2 abundance in the root of *rop2-1*/pROP2:GFP:ROP2 Arabidopsis treated with 0 (-GSNO) or 250 µM GSNO using anti-GFP antibody. (**C**) pROP2:GFP:ROP2 fluorescence in Arabidopsis root tips treated with 0 or 250 µM GSNO. (**D**) Binding efficiency of 6xHIS:ROP2 to GTP-agarose without (DMSO) or with the indicated concentrations of GSNO treatment. The percentages of input ROP2 bound to and eluted from GTP-agarose are shown. The data are average and standard deviation for three independent experiments with two technical repetitions each. Different letters sign significant difference at the *p* < 0.05 level based one one-way ANOVA and Duncan’s test.

It was investigated whether GSNO treatment could alter the expression/abundance of ROP2 in the root. Although the transcript level of the endogenous *ROP2* gene slightly, but significantly increased upon GSNO application, neither the transcript level nor the amount/fluorescence of the GFP:ROP2 protein controlled by the *ROP2* promoter in the *rop2-1* background changed in response to the treatment (Figure 3A–C).

To test whether the S-nitrosation of ROP2 might alter its biochemical properties, we tested the in vitro GTP-binding affinity of purified 6xHIS:ROP2 following its treatment with GSNO. However, no significant difference could be detected among the treated and untreated samples even at high GSNO concentrations (Figure 3D).

**Figure 4:**
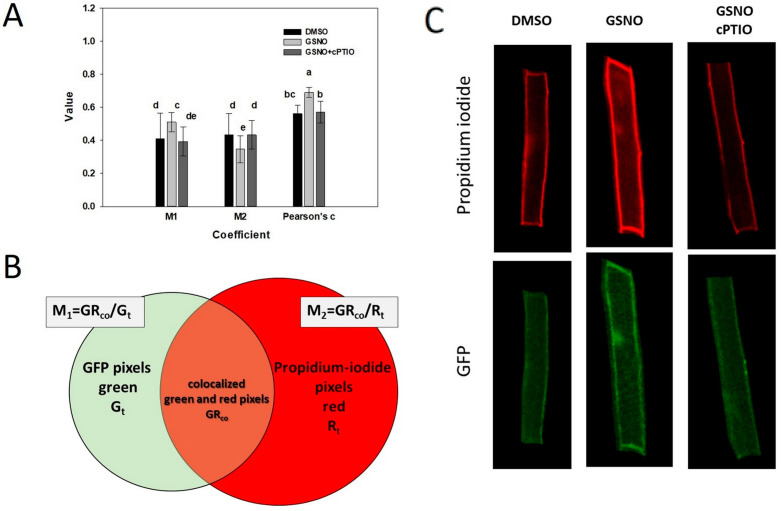
Effect of GSNO treatment on the intracellular localization of GFP:ROP2 expressed under the control of its own promoter in root cells. (**A**) Average values of Manders’ colocalization coefficients (M1 and M2) and Pearson’s coefficient. Seven-day-old Arabidopsis seedlings were treated 4 h in dark as indicated (DMSO as solvent control, 250 μM GSNO as NO donor, 800 μM cPTIO as NO scavenger) and further cultured for 20 h before fluorescent microscopy. Cell periphery was stained by propidium iodide (PI). Individual cell images in silico separated from whole stained root pictures with the aim of the ImageJ plugin JaCoP were used for localization studies [49]. Mander’s colocalization coefficients (M1, M2) and Pearson’s coefficient were calculated for 22-22 cells according to [50]. Statistical analysis was analysis of variance followed by Duncan’s test. Different letters mean significant difference at the *p* < 0.05 level. M1—ROP2 fraction at the cells’ periphery out of total ROP2 expressed; M2—the fraction of cell periphery that has ROP2; Pearson’s c—the overall correlation of green and red pixels in the cells. (**B**) The calculation of M1 and M2 coefficients. Gt—total amount of green pixels generated by the expression of GFP-tagged ROP2 in the region of interest (ROI) studied. Rt—total amount of red pixels in a ROI, generated by propidium–iodide molecules labelling the cell’s periphery. PI labels only whole cells with undamaged, intact membranes and only those cells were chosen as ROI. GRco—indicate green pixels colocalized with red pixels. (**C**) Example individual cell images used to calculate the coefficients shown in part A. Individual cell images were separated as ROI from whole stained roots using the ImageJ plugin, JaCoP [49].

GTP-bearing ROP GTPases exert their signaling function being attached to the inner surface of the plasma membrane, but are released into the cytoplasm after hydrolyzing the GTP. Cell surface cysteines have a role targeting the GTPase to specific membrane subdomains [48]. We evaluated, therefore, the potential effect of GSNO (cysteine nitrosation) on the intracellular distribution of the GFP-tagged ROP2 GTPase that was expressed under the control of its own promoter. For this, roots of pROP2:GFP:ROP2-expressing plants were stained with propidium iodide to mark the cells’ periphery. The degree of the overlap between green and red fluorescent pixels was calculated to determine the proximity of the GFP:ROP2 protein to the cell periphery (cortical region). Due to technical reasons, only epidermal cells of the transition and differentiation regions of the root could be studied (see Supplementary Materials and methods). It was observed that one day after the treatment of the roots, significantly more GFP:ROP2 protein was co-localized to the cell cortex (increased M1 coefficient); however, the co-localization was attributed to a smaller overlapping region (decreased M2 parameter) (Figure 4). Importantly, co-application of GSNO with the NO scavenger cPTIO prevented the reorganization of the cortical abundance of GFP:ROP2 supporting the NO-dependence of its cellular distribution at least in this cell type (Figure 4).

### 2.4. GSNO-Triggered PIN1 Depletion Is Dependent on ROP2 in the Arabidopsis Root Meristem

**Figure 5:**
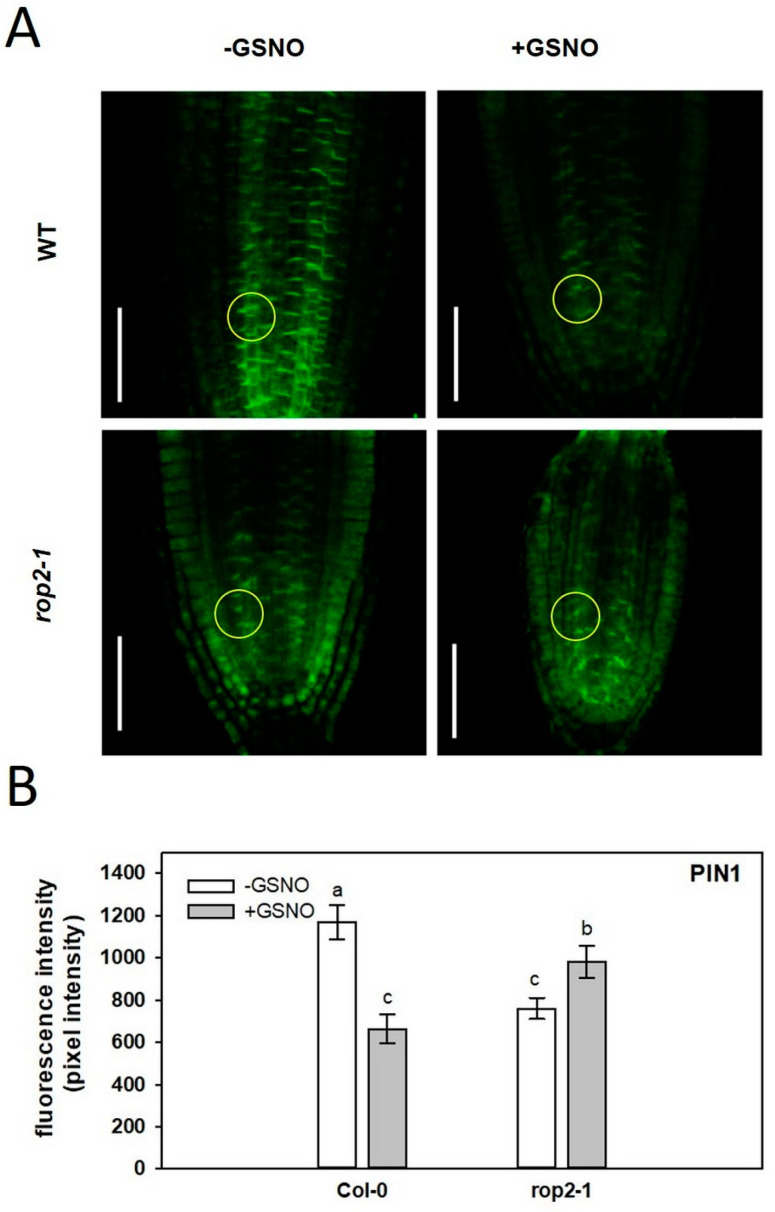
(**A**) Immunofluorescence localization of the PIN 1 protein in PR meristem of control (-GSNO) and 250 µM GSNO-treated wild type (WT) and *rop2-1* Arabidopsis seedlings. Bars = 50 µm. (**B**) Intensity of PIN1-associated whole-mount immunofluorescence in the PR meristem of wild-type and *rop2-1* Arabidopsis grown without or with 250 µM GSNO. Pixel intensities were measured in the stele in circle areas with 25 µm radii at 50 µm distance from the quiescent center cells (encircled). Different letters sign significant differences according to ANOVA and Duncan’s test (n = 20, *p* ≤ 0.05).

It has been reported that GSNO decreases the auxin maximum in the PR apex [16]. To investigate whether GSNO affects auxin transport in a ROP2-dependent way, localization of the PIN1 protein was examined via immunofluorescence in the apex of wild-type and *rop2-1* roots exposed to GSNO (Figure 5). In the PR meristem of Col-0, GSNO decreased PIN1 protein abundance, while in the *rop2-1* mutant, PIN1 level (stability) was increased in response to GSNO. It was also noted that PIN1 protein abundance was lower in the stele, but higher in the cortex of the ROP2-deficient root apices than in that of the wild-type. These observations indicate that GSNO disturbed the regulation of PIN1 abundance in the Arabidopsis root meristem dependent on the presence of ROP2.

## 3. Discussion

### 3.1. NO Effect on Root Shortening Depends on ROP2

NO has already been reported to reduce auxin transport and, concomitantly, root meristem activity in a PIN1 auxin efflux carrier-dependent manner [16]. We confirmed that in NO-treated wild-type Arabidopsis roots, the abundance of the PIN1 protein was considerably altered in the meristem region (Figure 5). This observation agreed with NO-induced moderate auxin accumulation in the meristem [16] and limited root growth (Figure 1). Interestingly, the NO treatment could not decrease neither the PIN1-dependent GFP fluorescence nor the root growth in the *rop2-1* mutant background. The fact that the *rop2-1* mutant roots exhibited a reduced response to NO treatment suggests that the negative effect of NO on root growth and PIN1 abundance is mediated by ROP2. In agreement, PIN1:GFP fluorescence at the root tips was lower in the *rop2-1* mutant than in the wild type (Figure 5). However, the absence of ROP2 did not result in limited root growth (Figure 1), indicating that the presence of S-nitrosated ROP2 in the root has more severe consequences than the absence of the protein.

One can imagine various scenarios as to how ROP2 is involved in this process. One possibility is that S-nitrosation of ROP2 influences PIN1 stability at the posttranslational level. It is known that the plasma-membrane-bound PIN auxin efflux carrier proteins undergo constitutive endocytosis and recycling during auxin-mediated cell polarity establishment [30,37,38]. Both endo- and exocytosis of several Arabidopsis PIN proteins have been shown to be regulated by ROP GTPases [30,37,38]. In the lobe region of leaf pavement cells, a positive feedback loop between PIN1-mediated auxin efflux and ROP2-inhibited PIN1 endocytosis was experimentally established [30,36]. Experimental evidence also indicated that ROPs control exocytic PIN1 recycling [37]. Our results can be explained if ROP2 have a role in the regulation of PIN1 abundance not only in the leaf, but also in the root. It has been already shown that in the root of Arabidopsis, the ROP6 GTPase negatively regulates clathrin-mediated endocytosis of PIN1 and PIN2 [30,38]. Interestingly, although ROP6 is also prone to be in vitro S-nitrosation similarly to ROP2 (Supplementary Figure S2A), it is not required for NO-dependent root shortening (Supplementary Figure S2B). This can be explained by the specific expression patterns (Supplementary Figure S3) and/or signaling partners of these two ROPs in the root.

The direct regulation of PIN1 protein recycling by the ROP2 GTPase requires that these two proteins are present in the same cells in the root meristem. Investigating the plant single-cell browser database [51] allowed the direct comparison of the expression pattern of the *ROP2*, *ROP6*, *PIN1,* and *PIN2* genes in the Arabidopsis root (Supplementary Figure S3). While the *PIN2* gene is widely and strongly expressed in the root including the meristem, high *PIN1* expression is restricted only to a few cells in this region. ROP2 is also widely expressed in all root cell types at a high level, while the expression of ROP6 is more restricted in some regions only to few cells in the meristem and the pericycle, for example. One can note the strong overlapping expression of ROP2 (but not ROP6) with PIN2 (cluster 3) and PIN1 (cluster 12) in the stem cell niche region of the meristem (Supplementary Figure S3). Therefore, the ROP2-dependent regulation of neither the PIN1 nor the PIN2 proteins can be excluded based on gene expression data in the Arabidopsis root meristem.

Another possibility is that NO decreases the transcription of the *PIN1* gene. *PIN1*transcription was reported to be rather sensitive to conditions affecting auxin transport and distribution [52]. Furthermore, there are experimental data showing that endogenous as well as exogenous NO influences the auxin transport pattern in the Arabidopsis root meristem in a PIN2 stability- and distribution-dependent manner [53,54]. Therefore, it is also possible that the NO-induced and PIN2-dependent auxin decrease in the root meristem feeds back to reduce PIN1 as well as PIN2 transcription. The synergistic interactions of PIN proteins involving the cross-regulation of PIN gene expression have been demonstrated [55]. The absence of ROP2 might interfere with this regulation in the NO-treated root, affecting either PIN1 or PIN2 stability and/or transcription.

The application of NO donors as well as the investigation of the NO-overproducing *cue1/nox1* Arabidopsis mutant, however, revealed that the NO-dependent decrease of PIN1-GFP fluorescence in the root was independent of the proteosome [16]. This implies that the direct targets of NO are not the PIN proteins affecting the auxin transport; but rather, auxin signaling affecting the expression of *PIN* genes and, so, the auxin transport. Our results implicate the ROP2 GTPase in this process. Interestingly, however, NO-mediated S-nitrosation of the auxin receptor TRANSPORT INHIBITOR RESPONSE 1 (TIR1) had a positive effect on auxin signaling [42]. Furthermore, auxin is known to regulate the endogenous NO levels [42,53,54]. Therefore, auxin and NO can be part of complex regulatory loops required to maintain root meristem functions [54]. At present, it is hard to define at what step the ROP2 GTPase is involved in this regulation. in planta identification of the S-nitrosated ROP2 GTPase, and its interaction with regulators and effectors could give insight into the NO-dependent control of ROP GTPase signaling. However, it is technically a rather challenging task due to the low cellular abundance of these signaling proteins. A hint for their potential posttranslational regulation via S-nitrosation can be obtained investigating NO-exposed purified proteins.

### 3.2. S-nitrosation of the AtROP2 GTPase Might Control Its Function

Cysteine S-nitrosation is the major reversible NO-dependent post-translational modification of proteins affecting various cellular functions [56,57]. In animal cells, several small GTP-binding proteins of the Ras superfamily including the Rho-type GTPases are among potential S-nitrosation targets [43]. Rho subfamily GTPases possess a cysteine, C18 (human Rac1 numbering), located at the end of the phosphoryl-binding P-loop (GXXXXGKS/TC) [44]. The C18 thiol in the GXXXXGK(S/T)C motif is solvent accessible and is, therefore, a potential target of ROS or reactive nitrogen species (RNS) [43].

Considering the observation that the effect of NO on primary root growth is ROP2 GTPase-dependent in Arabidopsis (Figure 1), the potential S-nitrosation of AtROP2 was investigated in vitro. It was found that the purified ROP2 protein could be S-nitrosated by GSNO (Figure 2). The primary structure of eukaryotic Rho GTPases is highly conserved, and plant ROP GTPases also have the GXXXXGK(S/T)C P-loop motif (Supplementary Figure S4A). Nevertheless, on-line protein S-nitrosation prediction tools indicated only C157 and C192 as potential S-nitrosation sites for Arabidopsis ROP2 (Supplementary Figure S4B).

S-nitrosation might alter the biochemical properties or the intracellular localization of the Rho-type GTPases. ROS and RNS were both found to promote guanine nucleotide dissociation from human Rac1, RhoA, and Cdc42 GTPases in vitro [44]. The redox sensitive C18 of Rac1 and its corresponding C20 of RhoA were shown to be required to mediate ROS or RNS action on nucleotide binding since their replacement by serine (Rac1 C18S) or alanine (RhoA C20A) residues, respectively, abolished the redox regulation [45,58]. Unfortunately, modification of the C20 residue of the AtROP2 GTPase cannot be easily investigated by mass spectrometry because the T19-D59 region contains neither tryptic cleavage site nor basic amino acids promoting ionization [59]. The production of C20Sm mutants can only provide information whether C20 is a redox-sensitive residue in AtROP2 similarly to C18 of Rac1 and C20 of RhoA. It has been previously demonstrated that the C20Sm mutation of the AtROP6 GTPase has wild-type GTP-binding and GTP-hydrolyzing activities [60]. In our experiments, AtROP2 exposed to even high concentrations of the NO-donor GSNO did not exhibit reduced binding to GTP agarose (Figure 3). Whether nitrosation of AtROP2 might affect GDP dissociation rather than GTP-binding, as described for animal Rho-type GTPases [43], needs to be further investigated.

As the online prediction tools indicated (Supplementary Figure S4B), the S-nitrosation of C157 and/or C192 of ROP2 can neither be excluded. The S-acylation of C157 was shown to regulate the membrane-association dynamics of the GTPase and specifically, its association with lipid rafts [60]. The C20Sm and C157Sm mutations preventing S-acylation interfered with cellular AtROP6 functions, including the regulation of polar growth, distribution of ROS, and endocytic recycling [60]. The C192 residue is part of the evolutionary conserved N-terminal CAAX motif required for the post-translational prenylation of Rho-type GTPases controlling their intracellular distribution via membrane associations [61]. It is to be noted that the S-nitrosation of neither C157 nor C192 equivalents has yet been experimentally validated in any Rho proteins. Nevertheless, it is theoretically possible that S-nitrosation of the same residues might interfere with or mimics S-acylation or prenylation of the GTPase, and can have respective functional consequences on its subcellular localization and function.

Our observations indicated that NO treatment resulted in altered GFP:ROP2 protein distribution in root cells. The treatment of the root with GSNO resulted in an increased accumulation ratio of the protein in the cortical region of the cell (Figure 4). One can suppose that the potential in planta S-nitrosation of AtROP2 either interferes with S-acetylation-dependent membrane association kinetics, directly or indirectly affecting nucleotide dissociation dynamics. One cannot exclude, however, the possibility that NO influences in planta AtROP2 mediated signaling via upstream regulatory factors, such as GEFs, GAPs, or GDIs.

## 4. Materials and Methods

### 4.1. Plant Material and Growth Conditions

Seeds of *Arabidopsis thaliana* (L.) Columbia-0, and the *rop2-1* (SALK_055328C) and *rop6-2* (SALK_ 091737C) mutant lines were obtained from the Nottingham Arabidopsis Stock Centre (NASC). The *rop2-1* knockout mutant was characterized previously in [46]; the *rop6-2* mutant by [38]. Transgenic pROP2:GFP:ROP2/*rop2-1* seeds were kindly provided by Prof. Lei Zhu (State Key Laboratory of Plant Physiology and Biochemistry, College of Biological Sciences, China Agricultural University, Beijing 100193, China) [47]. The seeds were surface sterilized with 70% (*v/v*) ethanol and 5% (*v/v*) sodium hypochlorite, and transferred to a half-strength Murashige and Skoog medium (1% (*w/v*) sucrose and 0.8% (*w/v*) agar). The Petri dishes were kept vertically in a greenhouse at a photo flux density of 150 µmol m^−2^ s^−1^ (12/12 day/night period) at a relative humidity of 55–60% and 25 ± 2 °C for 7 days.

### 4.2. Chemicals and Treatments

As NO donors, 50, 100, 250, 500, or 1000 µM S-nitroso-penicillamine (SNAP, Sigma-Aldrich, St. Louis, MO, USA), or S-nitrosoglutathione (GSNO, Sigma-Aldrich, St. Louis, MO, USA); and as an NO scavenger, 800 µM 2-(4-carboxyphenyl)-4,4,5,5-tetramethylimidazoline-1-oxyl-3-oxid (cPTIO, Sigma-Aldrich, St. Louis, MO, USA) was used. Stock solutions of SNAP, GSNO, or cPTIO (in DMSO) were added on the surface of the media containing the root system of 4-day-old seedlings through 2 mL sterile filters. In each Petri dish, 0.5 mL water (control) or SNAP/GSNO/cPTIO solutions were added, and those were kept horizontally for 2 h and lifted to the vertical position. Water or DMSO was added to the medium as controls. All the measurements were conducted 72 h later. The 250 µM concentration solutions of SNAP or GSNO were placed under 1900 μmol m^−2^ s^−1^ white light for 10 h [62] in order to obtain inactivated NO-donor solutions (iSNAP, iGSNO).

### 4.3. Microscopy-Based Analyses

PR lengths were measured manually using at least 20 plants per sample per experiment, and the rate of PR shortening (%) in the presence of treatments was calculated.

For intracellular localization analysis, Arabidopsis seedlings expressing pROP2:GFP:ROP2 were grown in a vertical position, under 8 h light/16 h dark, 220C/210C light and temperature cycles. Seven-day-old seedlings were treated for 4 h in the dark with either DMSO as a solution control, or 250 μM GSNO as an NO donor with or without 800 μM cPTIO as an NO scavenger. After treatment, the plates were placed back into the plant chamber in vertical position and the seedlings were grown for 20 more hours. Prior to imaging, roots were stained with 10 µg/mL propidium iodide for one minute; then, were washed once with distilled water and were prepared on microscopic slides. Optical sections of roots were prepared with a Zeiss LSM 880 (Carl Zeiss, Jena, Germany) confocal laser scanning microscope. Propidium iodide was excited by a 488 nm diode laser, and the emission was detected between 620 and 700 nm wavelengths. GFP fluorescence was excited by a 488 nm diode laser and was measured below 555 nm. Images taken were used in colocalization studies performed with the ImageJ plugin, JaCoP [49]. Mander’s colocalization coefficients (M1, M2) and Pearson’s coefficient were calculated for several cells according to [50]. Image processing is exemplified in the Supplementary Material and methods.

### 4.4. Detecting NO in Plant Roots and Its Quantification in Donor Solutions

Nitric oxide levels in Arabidopsis root tips were analysed by 4-amino-5-methylamino-2′,7′-difluorofluorescein diacetate (DAF-FM DA). Seedlings were incubated in 10 µM DAF-FM DA (prepared in 10 mM Tris-HCl, pH 7.4) for 30 min at room temperature in darkness and washed two times in buffer prior to microscopic analysis. Root tips were observed under a Zeiss Axiovert 200M microscope (Carl Zeiss, Jena, Germany) using filter set 10 (exc.: 450–490 nm; em.: 515–565 nm), and fluorescent intensities were measured by Axiovision Rel. 4.8 software. In each experiment, at least 10 seedlings were observed.

Measurement of NO concentration in GSNO and SNAP solutions was performed using an NO-sensitive electrode (ISO-NOP, 2 mm, World Precision Instruments Inc., Sarasota, FL, USA). Two mL of SNAP or GSNO (both at 250 µM concentration) solutions were prepared and measured immediately. Data were recorded at different time points (0, 15, 30, 45, 60, 90, 120, 360, 480 min). The constant mixing of the solutions was ensured by a magnetic stirrer. NO concentration (nM) was calculated from a standard curve created by the decomposition of SNAP in the presence of copper [63].

### 4.5. qRT-PCR Analysis

The expression rate of AtROP2 gene was determined by quantitative real-time reverse transcription-PCR (RT-qPCR). RNA was purified from 90 mg root tissue by using a NucleoSpin RNA Plant mini spin kit (Macherey–Nagel) according to the manufacturer’s instruction. An additional DNase digestion was applied (by using a DNA Clean and Concentrator Kit from Zymo Research and DNase I from Thermo Scientific), and cDNA was synthetized using RevertAid reverse transcriptase (Thermo Scientific). Primers were designed for the selected coding sequences using the Primer3 software; the primers used are listed in Table S1. The expression rate was monitored using the SYBR Green PCR Master Mix (Thermo Scientific) as described by Gallé et al. [64]. Data analysis was performed using qPCRsoft3.2 software (Analytik Jena AG, Jena, Germany). ACTIN2 (At3g18780) and GAPDH2 (At1g13440) genes were used as internal controls.

### 4.6. Western Blot Analysis of GFP:ROP2 Protein Abundance

Western blot analysis was carried out using 1:2000 dilution of an anti-GFP rat monoclonal antibody (3H9; Chromotek, Planegg-Martinsried, Germany) and standard procedures. In all, 20 µg of total protein extracts were separated on 12% SDS–polyacrylamide gels. After separation, the proteins were transferred onto PVDF membranes (Immobilon-P, Merck Millipore, Merck KGaA, Darmstadt, Germany). The blocking solution was TBS (150 mM NaCl, 50 mM Tris-HCl, pH 7.5) with 0.2% Tween and 5% non-fat milk. Horseradish peroxidase-conjugated anti-IgG (H,L) rat secondary antibody (BI2411, Abliance, Compiègne, FRANCE) was used at 1:10,000 dilution. Immunoreactive bands were visualized using the Immobilon Western chemiluminescent HRP substrate (WBKLS0500, Merck Millipore) and X-ray films (Super RX-N, Fuji Medical, Tokyo, Japan).

### 4.7. Molecular Cloning

The wild-type ROP2 sequence was amplified by polymerase chain reaction (PCR) using EcoRI-site extended N-terminal and XhoI-site extended C-terminal oligonucleotide primers, and the PHUSION II high-fidelity DNA polymerase (Thermo Fisher Scientific, Waltham, MA, USA). The gel-purified PCR product was digested with EcoRI and XhoI, repurified, and cloned into the EcoRI/XhoI sites of the pET28a vector (Merck Millipore). The cloned fragments were sequenced.

For the used oligonucleotide sequences, see Supplementary Table S1.

### 4.8. Recombinant Protein Expression

The protein expression constructs were transformed into the BL21 (DE3) Rosetta strain (Merck Millipore). The selected bacterial cells carrying the constructs were inoculated into 30 mL liquid Luria-Bertani (LB) media containing the kanamycin sulphate antibiotic (30 μm/mL), 2% (*v/v*) glucose, and were grown overnight at 37 °C. The following day, cells were diluted to 200 mL with liquid LB media containing the same antibiotics, 0.4% (*v/v*) glucose, and grown until OD600 reached the 0.6 value. Protein expression was induced using 1 mM Isopropyl β- d-1-thiogalactopyranoside (IPTG; Thermo Fisher Scientific); then, the cells were incubated at 37 °C for 2 h with constant shaking. Expressed proteins were then purified with the HIS-Select nickel affinity gel (Sigma-Aldrich, St. Louis, MO, USA) following the manufacturer’s instructions. Purified proteins were concentrated using Amicon Ultra 0.5 mL filters (Merck Millipore); and then, stored in 40% (*v/v*) glycerol at −20 °C for later use.

### 4.9. Biotin Switch Assay

The biotin switch technique (BST; Ref. [65]) was used to analyse the S-nitrosation of proteins. In total, 9 µg of recombinant AtROP2 wild-type of Cys mutants in HEN buffer (100 mM Hepes-NaOH pH 7.7, 1 mM EDTA, 0.1 mM neocuproine) were treated with 250 µM GSNO in a final volume of 150 µL for 30 min at RT in the dark with intermittent inverting of the tubes. Free thiols were blocked with 50 mM N-ethylmaleimide (NEM) in the presence of 2% (*w/v*) SDS for 20 min at 37 °C in the dark (vortexing every 5 min). Excess GSNO and NEM was removed by acetone precipitation (20 min, −20 °C); the protein pellets were washed with ice-cold acetone (10,000g for 10 min at 4 °C), air-dried, and then, resuspended in 20 µL of HENS buffer (HEN supplemented with 1% (*w/v*) SDS). The labeling reaction was started by the reduction of S-nitrosothiol groups with sodium ascorbate (1 mM final concentration). Control treatment was performed with water. The nascent reduced thiols, which were originally S-nitrosated, were then biotinylated with 1 mM biotin-HPDP. The samples were incubated for 1 h at RT. After acetone precipitation, the protein pellets were washed with ice-cold acetone (10,000 g for 10 min at 4 °C), air-dried, and then, resuspended in 15 µL non-reducing SDS-PAGE sample buffer (0.06 M Tris-HCl, pH 6.8, 10% (*v/v*) glycerol, 0.008% (*w/v*) bromophenol blue) and separated on a 10% SDS-PAGE gel without boiling prior to loading. The proteins were transferred onto a nitrocellulose membrane and stained with Ponceau S. Afterwards, biotinylated proteins were detected with an anti-biotin antibody conjugated with alkaline phosphatase.

### 4.10. In Vitro S-nitrosation and GTP-Binding Assay

S-nitrosation was performed with increasing GSNO concentration (0, 50, 100, 250, 500, and 1000 μM dissolved in DMSO). Equal amounts of recombinant 6xHIS:AtROP2 protein were S-nitrosated at room temperature in the dark for 45 min. Meanwhile, immobilized gamma-amino-hexyl-GTP-agarose beads (Jena Bioscience, Jena, Germany; AC-117S) were preequilibrated with GTP-binding buffer (20 mM TRIS pH = 7.5, 150 mM NaCl, 15 mM MgCl_2_ supplied with 1 mM PMSF). After nitrosation, protein solutions were diluted 17-fold with a binding buffer and the same amount of GTP-agarose was supplied to each protein vial. Binding was performed in the dark at 8 °C in a cold room with mild rotation of solutions for 3 h. Flowthrough was removed by mild centrifugation (1 min, 3000 rpm) and the beads were washed several times with binding buffer. Elution of the bound proteins was achieved by 5 min incubation of the beads at 95 °C in Laemli protein loading buffer. Proportional volumes of samples from each working step (after nitrosation: input, flowthrough—not bound, elution—GTP bound) were taken and loaded on 15% SDS-acrylamide gel. After electrophoresis, gels were stained with Coomassie blue for 20 min; destained with 10% ethanol–10% acetic acid solution in repeated steps until background was cleared. Gels were scanned, and the ImageJ software [66] was used for densitometric measurements and binding capacity calculations. Three independent, but similar experiments were performed; and for each, two technical repetitions of the SDS-PAGEs were conducted.

### 4.11. S-nitrosation Site Prediction and In Silico Gene Expression Analysis

The AtROP2 protein sequence (Q38919) was extracted from UNIPROT (www.uniprot.org, accessed 2 February 2023) and submitted to protein S-nitrosylation/S-nitrosation prediction tools using high-threshold search parameters. 

The following tools were used: GPS-SNO 1.0 (http://sno.biocuckoo.org/, [67], last accessed 2 February 2023); DeepNitro (http://deepnitro.renlab.org/, [68], last accessed 2 February 2023); iSNO-AAPair (http://app.aporc.org/iSNO-AAPair/, [69], last accessed 2 February 2023).

For cell-specific gene expression comparison, the available online Plant Single Cell Browser tool was used (https://www.zmbp-resources.uni-tuebingen.de/timmermans/plant-single-cell-browser/, last accessed 20 January 2023) [51].

### 4.12. Whole-Mount Immunolocalization of PIN1

PIN1 proteins were immunolocalized in the PR meristem of seven-day-old Col-0, *rop2-1* Arabidopsis according to [70] with slight modifications. Whole seedlings were fixed in a solution containing 4% formalin, 1x microtubule-stabilizing buffer (MTSB), and 0.1% Triton X-100. Samples were vacuum infiltrated two times for 2 min each, and incubated at room temperature for 45 min and at 37 °C for additional 15 min. Fixed seedlings were treated with warm methanol for 15 min, washed several times, and exposed to enzyme treatment (0.3% driselase, 0.15% macerozyme, 5 mM MES, pH 5.3) at 37 °C for 20 min. This step was followed by permeabilization by 10% DMSO, 3% IGE-PAL in 1x MTSB for 20 min at 37 °C. Prior to antibody labeling, samples were blocked by 2% BSA (in 1x MTSB pH 8.2) for 30 min. Seedlings were incubated with anti-PIN1 mouse monoclonal primary antibody (1:30) for 2 h in darkness, washed with MTSB, and then, incubated with a secondary antibody (Alexa Fluor 488 goat anti-mouse IgG, 1:500) for an additional 1 h. As a final step, seedlings were transferred to microscopic slides in a mounting medium (Fluoromount G). Under the Zeiss Axioscope 200-C stereomicroscope (Carl Zeiss, Jena, Germany), shoots and upper root zones were removed from the seedlings. Slides were kept at 4 °C until microscopic analysis, which was performed using an Olympus LSM 700 (Olympus, Tokyo, Japan) laser scanning microscope. Relative fluorescent intensities were determined using Olympus Fluoview FV100 software. Circles with 25 µm radii were placed at a 50 µm distance from the quiescent center cells.

### 4.13. Statistical Analysis

All the experiments were conducted at least three times. Results are expressed as mean ± SE. Multiple comparison analyses were performed with SigmaStat 12 software using an analysis of variance (ANOVA, *p* < 0.05) and Duncan’s test.

## 5. Conclusions

In this paper, we summarize observations that indicate the role of S-nitrosation in the regulation of the cellular function of plant ROP GTPases. More specifically, we found that the Arabidopsis ROP2 GTPase can be in vitro nitrosated, and the ROP2 loss-of-function mutant roots have reduced sensitivity towards NO treatment. These observations highlight the possibility that similarly to animal Rho-type GTPases, plant ROPs might also be directly controlled by the cells’ redox/nitrosation status via sensitive cysteines. Further specifying the link between ROP GTPase- and NO-mediated signaling pathways might open a new research direction.

## Data Availability

Not applicable.

## Supplementary Materials

The following supporting information can be downloaded at: https://www.mdpi.com/article/10.3390/plants12040750/s1, Supplementary Materials and Methods: Examples of image analysis Supplementary Figure S1: Nitric oxide levels in Arabidopsis roots; Figure S2: AtROP6 is not involved in NO-induced root shortening although can be nitrosated in vitro; Figure S3: In silico expression analysis of *PIN1*, *PIN2*, *ROP2*, and *ROP6* genes in the Arabidopsis root meriste; Supplementary Figure S4: Potential nitrosation sites of AtROP2; Supplementary Table S1: Oligonucleotide primers used in the study; Supplementary Table S2: Concentrations of liberated NO in 250 µM GSNO or SNAP solutions at different time points.
