# Supplementary material for: The ROP2 GTPase Participates in Nitric Oxide (NO)-Induced Root Shortening in Arabidopsis
Source: Plants (Basel). 2023 Feb 8;12(4):750. doi: 10.3390/plants12040750 (PMC9964108; doi:10.3390/plants12040750)
Supplement: Supplementary file 1 [file plants-12-00750-s001.zip › Figure S1.pdf]

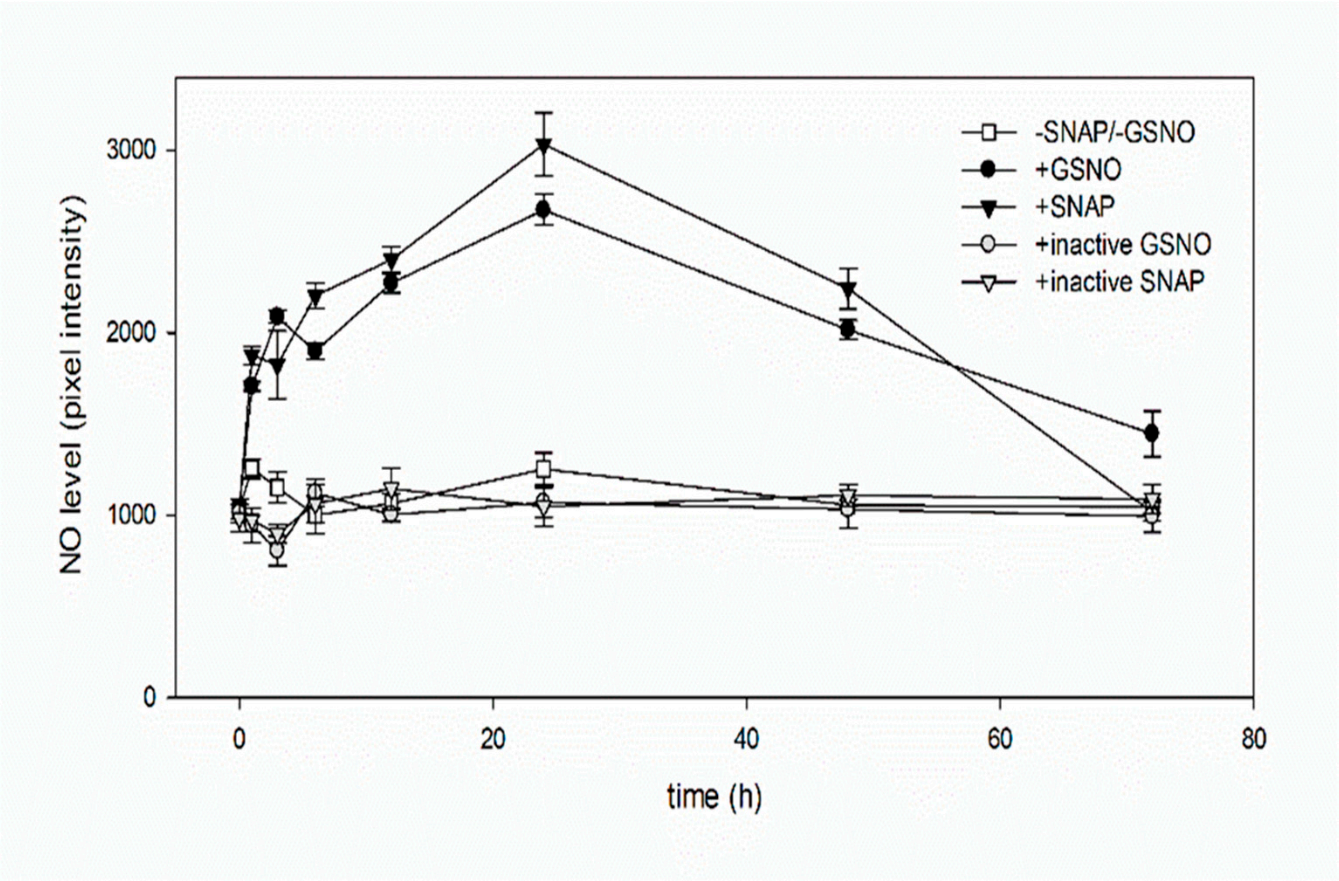


**Fig. S1** Nitric oxide levels in Arabidopsis roots. Nitric oxide levels (determined as pixel intensity of DAF-FM-derived fluorescence) in the root tips of wild-type Arabidopsis treated with 250 µM SNAP or 250 µM GSNO. Samples were taken 0, 1, 3, 6, 12, 24, 48 and 72 hours after treatments. Untreated plants served as controls (-SNAP/-GSNO) (n=10). The 250 µM concentration solutions of SNAP or GSNO were placed under 1900 µmol m-2 s-1 white light for 10 h (according to Wodala and Horváth 2008) in order to get inactivated NO donor solutions.
