# Supplementary material for: The ROP2 GTPase Participates in Nitric Oxide (NO)-Induced Root Shortening in Arabidopsis
Source: Plants (Basel). 2023 Feb 8;12(4):750. doi: 10.3390/plants12040750 (PMC9964108; doi:10.3390/plants12040750)
Supplement: Supplementary file 1 [file plants-12-00750-s001.zip › Figure S2.pdf]

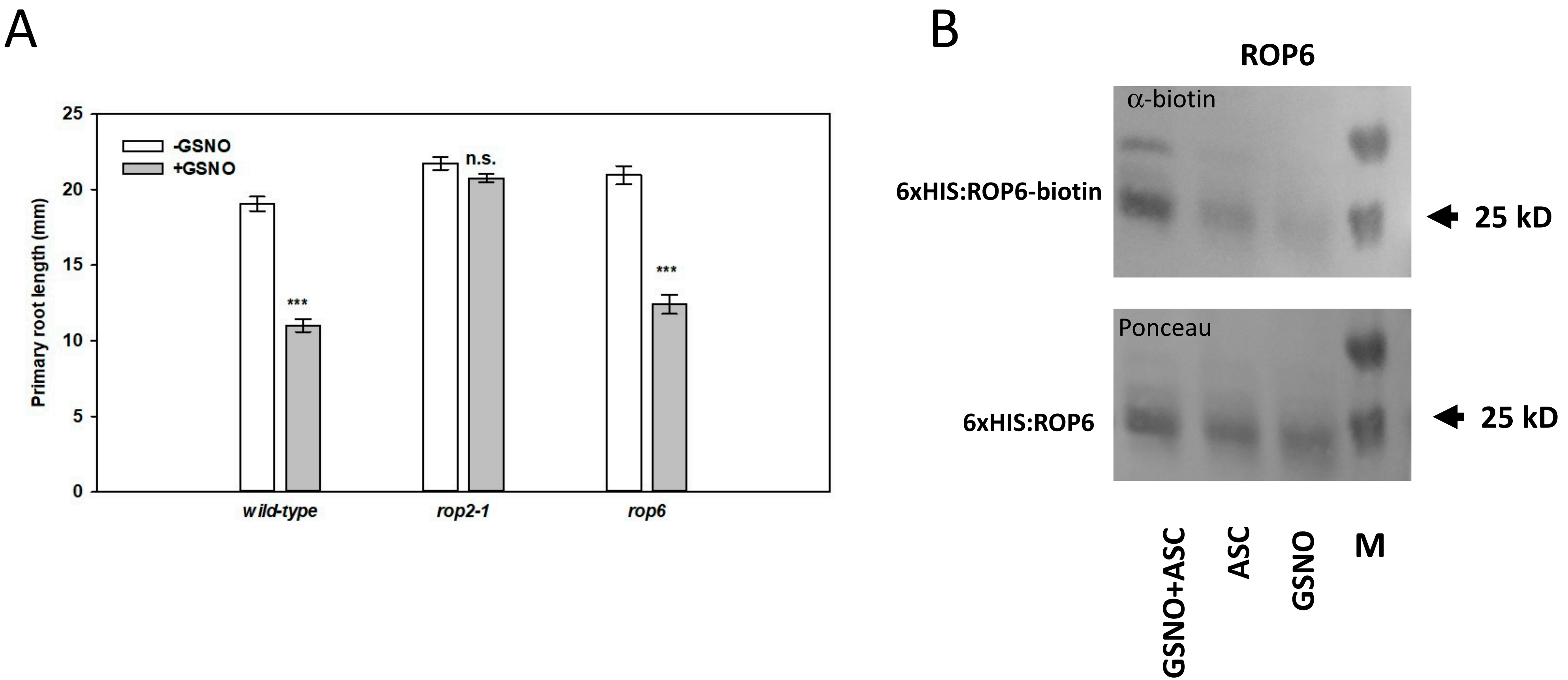

Fig. S2 AtROP6 is not involved in NO-induced root shortening although can be nitrosated *in vitro*. A) Comparison of the effect of 250 μM GSNO on root length of wild type, *rop2-1*, and *rop6-2* mutant Arabidopsis seedlings. Results are expressed as mean ± SE. Multiple comparison analyses were performed with SigmaStat 12 software using analysis of variance and Duncan's test. n.s. – not significant; *** - significant at the P<0.05 level. B) In vitro S-nitrosation of GSNO-treated 6xHIS:ROP6 protein as analysed by the "biotin switch" method (upper picture). The absence of ascorbate (Asc) or GSNO served as control. Ponceau S staining is shown as loading control (lower picture). M – molecular mass standard
