# Supplementary material for: The ROP2 GTPase Participates in Nitric Oxide (NO)-Induced Root Shortening in Arabidopsis
Source: Plants (Basel). 2023 Feb 8;12(4):750. doi: 10.3390/plants12040750 (PMC9964108; doi:10.3390/plants12040750)
Supplement: Supplementary file 1 [file plants-12-00750-s001.zip › Figure S3.pdf]

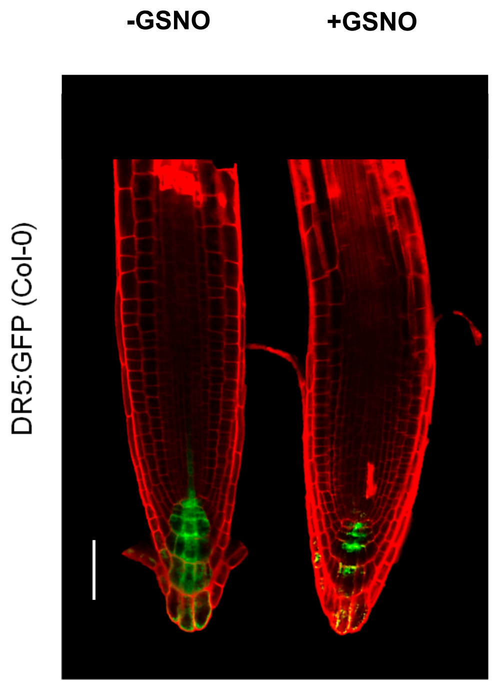

Fig. S3 In situ expression of DR5:GFP in PR apex of Arabidopsis (Col-0) root apex in the absence (-) or in the presence (+) of 250 µM GSNO. Bar= 50 µm.
