# Supplementary material for: The ROP2 GTPase Participates in Nitric Oxide (NO)-Induced Root Shortening in Arabidopsis
Source: Plants (Basel). 2023 Feb 8;12(4):750. doi: 10.3390/plants12040750 (PMC9964108; doi:10.3390/plants12040750)
Supplement: Supplementary file 1 [file plants-12-00750-s001.zip › Figure S4.pdf]

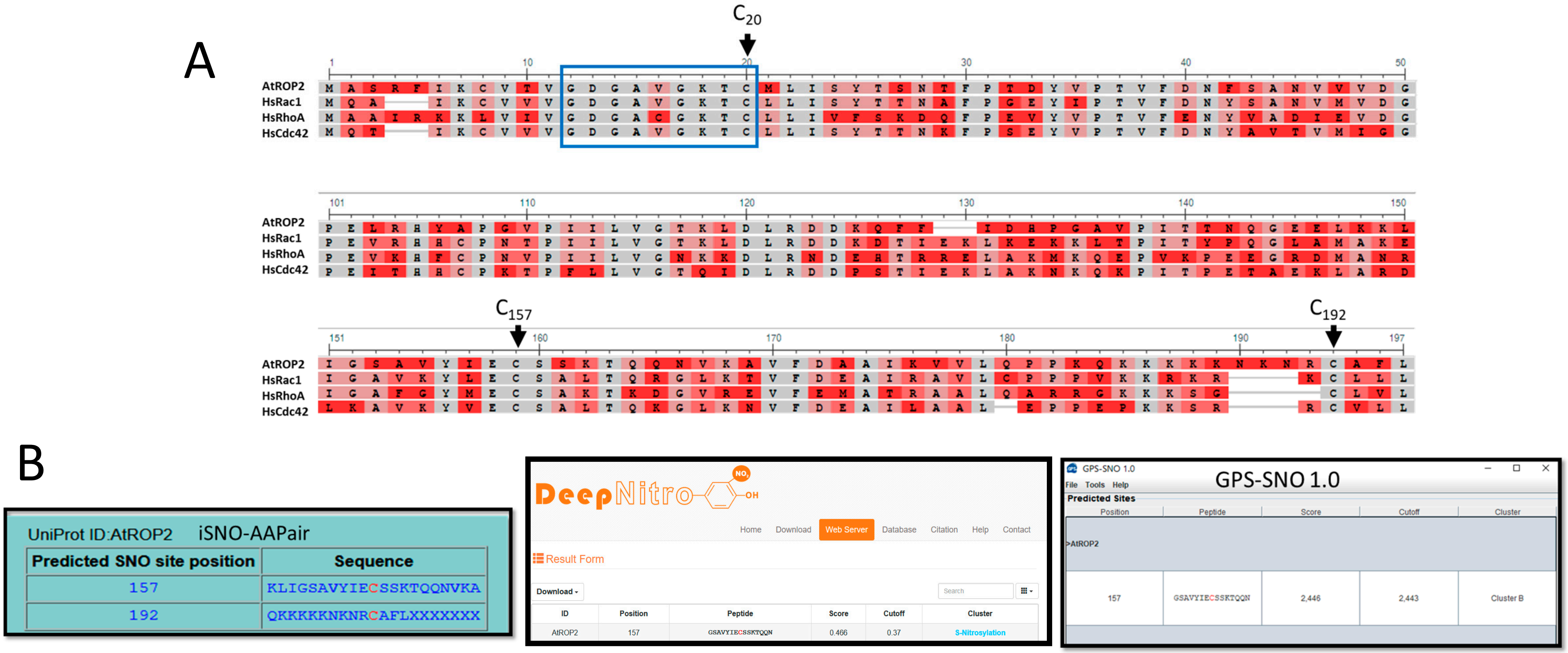

Fig. S4 Potential nitrosation sites of AtROP2. A) Aligment of RHO GTPase sequences highlighting the phosphoryl-binding P-loop motif GXXXXGK(S/T)C (boxed) and the evolutionary conserved C_{20}, C_{157}, and C_{192} cysteine residues of AtROP2. At – Arabidopsis thaliana; Hs – Homo sapiens. B) The AtROP2 protein sequence was submitted to three protein S-nitrosylation/S-nitrosation prediction tools using high threshold search parameters. The obtained results are shown. The following tools were used:

- iSNO-AAPair (http://app.aporc.org/iSNO-AAPair/);
- DeepNitro (http://deepnitro.renlab.org/);
- GPS-SNO 1.0 (http://sno.biocuckoo.org/).
