# Supplementary material for: The ROP2 GTPase Participates in Nitric Oxide (NO)-Induced Root Shortening in Arabidopsis
Source: Plants (Basel). 2023 Feb 8;12(4):750. doi: 10.3390/plants12040750 (PMC9964108; doi:10.3390/plants12040750)
Supplement: Supplementary file 1 [file plants-12-00750-s001.zip › Table S1.pdf]

Table S1. Oligonucleotide primers used in the study

**qRT-PCR analízis**			
Gene name	Locus identifier	Forward primer (5' to 3')	Reverse primer (5' to 3')
Actin2	At3g18780	GGTAACATTGTGCTCAGTGGTGG	AACGACCTTAATCTTCATGCTGC
GAPDH2	At1g13440	AATGGAAAATTGACCGGAATGT	CGGTGAGATCAACAACTGAGACA
ROP2	At1g20090	GCAGAACGTGAAGGCAGTGT	AACGCGCAACGGTTCTTATTC
**Cloning**			
Gene name	Locus identifier	Forward primer 5' to 3' sequence (added **EcoRI site** at 5')	Reverse primer 5' to 3' sequence (added **XhoI site** at 5')
ROP2	At1g20090	AT**GAATTC**ATGGCGTCAAGGTT TATAAAGTG	TC**CTCGAG**CAAGAACGCGCAACG GTTC
