# Supplementary material for: The ROP2 GTPase Participates in Nitric Oxide (NO)-Induced Root Shortening in Arabidopsis
Source: Plants (Basel). 2023 Feb 8;12(4):750. doi: 10.3390/plants12040750 (PMC9964108; doi:10.3390/plants12040750)
Supplement: Supplementary file 1 [file plants-12-00750-s001.zip › Table S2.pdf]

Table S2 Concentrations of liberated NO in 250 μM GSNO or SNAP solutions at different time points.

	released NO (nM)	
**Time (min)**	**250 μM GSNO**	**250 μM SNAP**
0	0.15 ± 0.04	0.19 ± 0.009
15	224.00 ± 5.29	151.35 ± 7.29
30	170.57 ± 4.28	626.65 ± 8.06
45	157.28 ± 7.02	625.28 ± 11.33
60	152.23 ± 6.49	598.45 ± 12.40
90	119.21 ± 10.11	538.37 ± 19.45
120	125.17 ± 5.89	567.95 ± 3.59
180	100.41 ± 9.19	474.86 ± 14.65
360	53.40 ± 6.77	403.77 ± 18.49
